# Case Report: Iparomlimab and Tuvonralimab-based therapy for epithelioid malignant peritoneal mesothelioma: a three-case series

**DOI:** 10.3389/fonc.2026.1914733

**Published:** 2026-09-03

**Authors:** Zhijin Xu, Lehe Zhu, Ying Fang, Yiyan Jiang

**Affiliations:** Department of Radiotherapy and Chemotherapy, The First Affiliated Hospital, Wenzhou Medical University, Wenzhou, School of The First Clinical School of Medicine (School of Information and Engineering), Wenzhou Medical University, Zhejiang, China

**Keywords:** antiangiogenic therapy, case report, CTLA-4, epithelioid malignant peritoneal mesothelioma, Iparomlimab, PD-1, QL1706, Tuvonralimab

## Abstract

**Background:**

Epithelioid malignant peritoneal mesothelioma (EMPM) is a rare and aggressive malignancy of serosal origin. Cytoreductive surgery combined with hyperthermic intraperitoneal chemotherapy is generally reserved for carefully selected patients in whom adequate cytoreduction appears feasible, while evidence supporting systemic immunotherapy remains limited for patients with unresectable disease or those unable to tolerate intensive treatment. Iparomlimab and tuvonralimab (QL1706) is a fixed-ratio anti-PD-1/anti-CTLA-4 MabPair formulation that has shown preliminary antitumor activity in several advanced solid tumors, but clinical experience in EMPM remains scarce.

**Case presentation:**

We retrospectively describe three patients with EMPM diagnosed through integrated clinicopathologic assessment who received QL1706 either as monotherapy or in combination with an antiangiogenic agent. Treatment response was assessed by the treating team on serial computed tomography (CT) using Response Evaluation Criteria in Solid Tumors version 1.1 (RECIST 1.1) as the evaluation framework.

**Results:**

Case 1 was a 53-year-old woman with a programmed death-ligand 1 (PD-L1) combined positive score (CPS) of 10. Diffuse peritoneal disease persisted after surgery and three sessions of postoperative catheter-based hyperthermic intraperitoneal chemotherapy. QL1706 plus bevacizumab subsequently produced stable disease with symptomatic improvement for approximately 7 months. Case 2 was an 89-year-old man with a PD-L1 CPS of 0 who was considered unsuitable for surgery or intensive chemotherapy. QL1706 plus dose-reduced lenvatinib achieved a partial response that remained ongoing for more than 10 months. Case 3 was a 70-year-old man with a PD-L1 CPS of 55 and pre-existing nephrotic syndrome. QL1706 monotherapy induced a rapid partial response, but treatment was discontinued because of worsening proteinuria and edema; the disease subsequently progressed, with a new intracranial lesion radiologically suspicious for metastasis.

**Conclusion:**

QL1706-based therapy achieved disease control in all three patients with EMPM who had limited conventional treatment options, including a durable response in an elderly patient with PD-L1-negative disease and rapid tumor regression with QL1706 monotherapy. The contrasting clinical courses suggest that overall benefit depends not only on tumor sensitivity but also on the accompanying treatment strategy and baseline organ function.

## 1 Introduction

Epithelioid malignant peritoneal mesothelioma (EMPM) commonly presents with diffuse peritoneal dissemination, omental involvement, ascites, and nonspecific abdominal symptoms. Recent consensus recommendations continue to regard cytoreductive surgery (CRS) combined with hyperthermic intraperitoneal chemotherapy (HIPEC) as an important treatment option for carefully selected patients and emphasize multidisciplinary assessment by teams experienced in peritoneal surface malignancies. Extensive disease, advanced age, impaired performance status, or major comorbidities may preclude locoregional treatment (1, 2).

Because large prospective studies in peritoneal mesothelioma are lacking, systemic treatment is partly informed by evidence from malignant pleural mesothelioma. CheckMate 743 established first-line nivolumab plus ipilimumab for unresectable pleural mesothelioma, while IND.227 showed improved outcomes with pembrolizumab added to platinum-pemetrexed chemotherapy (3, 4). In peritoneal mesothelioma, retrospective immune checkpoint inhibitor cohorts, a prospective study of atezolizumab plus bevacizumab, a case report of nivolumab plus ipilimumab, and a recent French real-world series have all documented clinically meaningful responses in selected patients, although no reliable predictive biomarker has been established (5–8).

QL1706 is a single fixed-ratio MabPair formulation comprising an anti-PD-1 IgG4 antibody and an anti-CTLA-4 IgG1 antibody, with differential pharmacokinetics designed to shorten systemic exposure to the CTLA-4 component. Early-phase studies and subsequent long-term follow-up have shown antitumor activity across several advanced solid tumors, with 5 mg/kg every 3 weeks used as the principal study dose (9–11). This formulation differs from separately administered PD-1 and CTLA-4 antibodies, although no direct comparative evidence has demonstrated superiority over established dual-checkpoint regimens.

Here, we describe the radiographic responses, treatment duration, and clinically relevant safety considerations in three patients with EMPM whose conventional treatment options were limited, thereby adding early clinical experience with QL1706 in this rare disease.

## 2 Case presentation

### 2.1 Case 1

**Figure 1:**
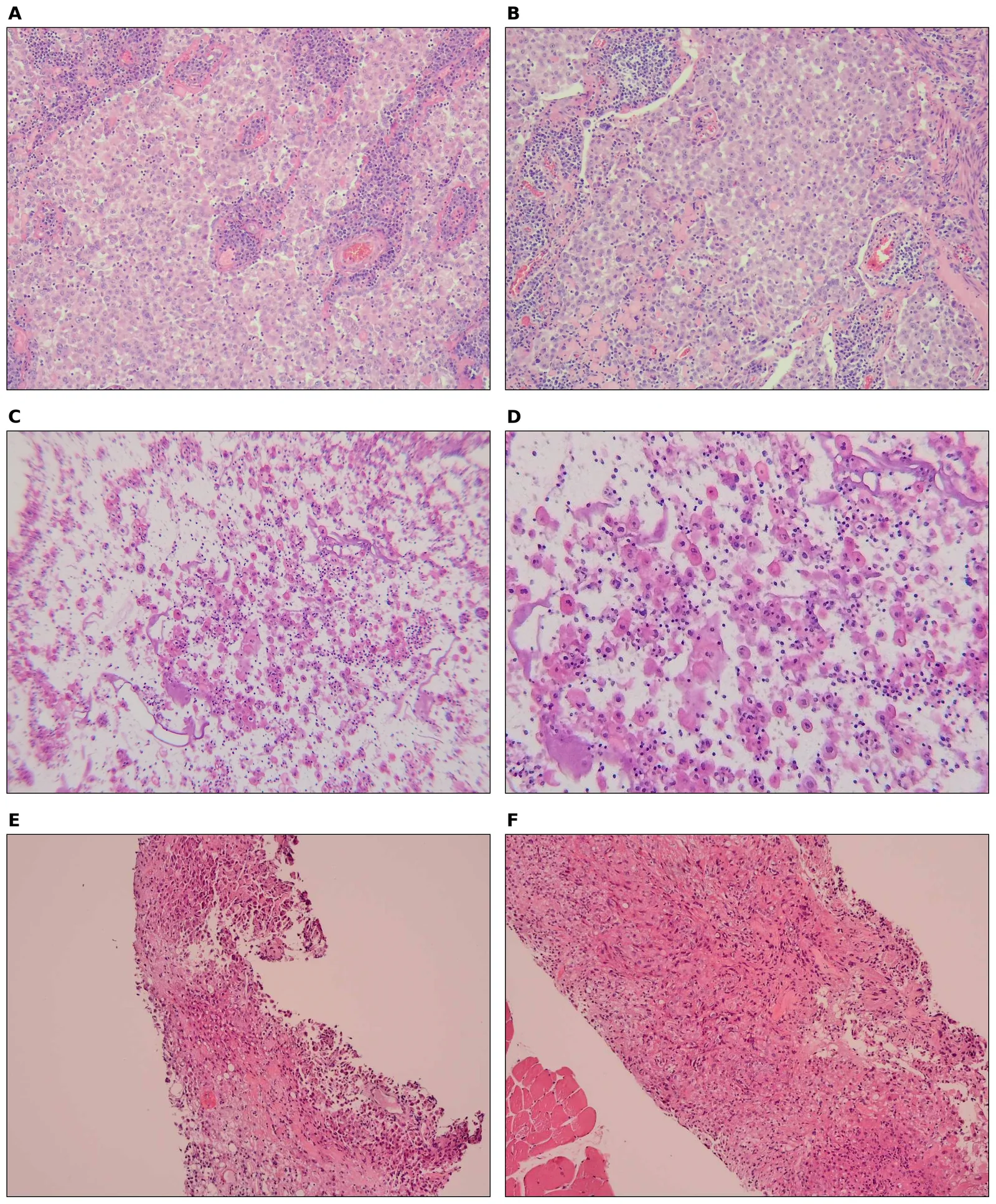
Representative pathologic images from the three patients. **(A, B)** Pelvic/peritoneal lesions from Case 1. **(C, D)** Ascitic-fluid cell-block specimens from Case 2; panel D is a higher-magnification view of the central region in panel **(C, E, F)** Core-needle biopsy of the abdominal mass from Case 3. All panels show hematoxylin and eosin staining.

A 53-year-old woman underwent laparoscopic total hysterectomy, bilateral salpingo-oophorectomy, and pelvic adhesiolysis for a pelvic mass. Intraoperative findings included abundant gelatinous ascites, a mass measuring approximately 4 cm in the rectouterine pouch, and scattered peritoneal nodules measuring approximately 0.5 cm. The resected pelvic and peritoneal lesions showed epithelioid morphology (**Figures 1A, B**). The tumor cells were positive for calretinin, CK5/6, D2-40, and CK7 and negative for PAX8, estrogen receptor, progesterone receptor, SATB2, and CDX2. Together with the morphology, this immunophenotype established the diagnosis of EMPM. The Ki-67 proliferation index was approximately 10%-15%, and the pathology report documented a PD-L1 CPS of 10. No additional tumor molecular testing was performed.

Approximately 6 weeks later, repeat laparoscopy revealed large-volume ascites, numerous miliary peritoneal implants, and diffuse omental involvement; no definite implants were seen on the surfaces of the liver or spleen. Pelvic adhesiolysis was performed, and intraperitoneal access and drainage catheters were placed. Systematic cytoreduction was not undertaken. The operative record did not include a formal 13-region Peritoneal Cancer Index (PCI), and the available information was insufficient for reliable retrospective reconstruction. Because formal CRS was not performed, a completeness of cytoreduction (CC) score was not applicable. From February 19 to 21, 2025, the patient completed three sessions of postoperative catheter-based hyperthermic intraperitoneal chemotherapy. Cisplatin 60 mg was used during the first and third sessions and pemetrexed 1 g during the second. Each session involved circulation of 2,500 mL of perfusate for 60 minutes, with inlet and outlet temperatures of 43.5 °C and 42.5 °C, respectively.

**Figure 2:**
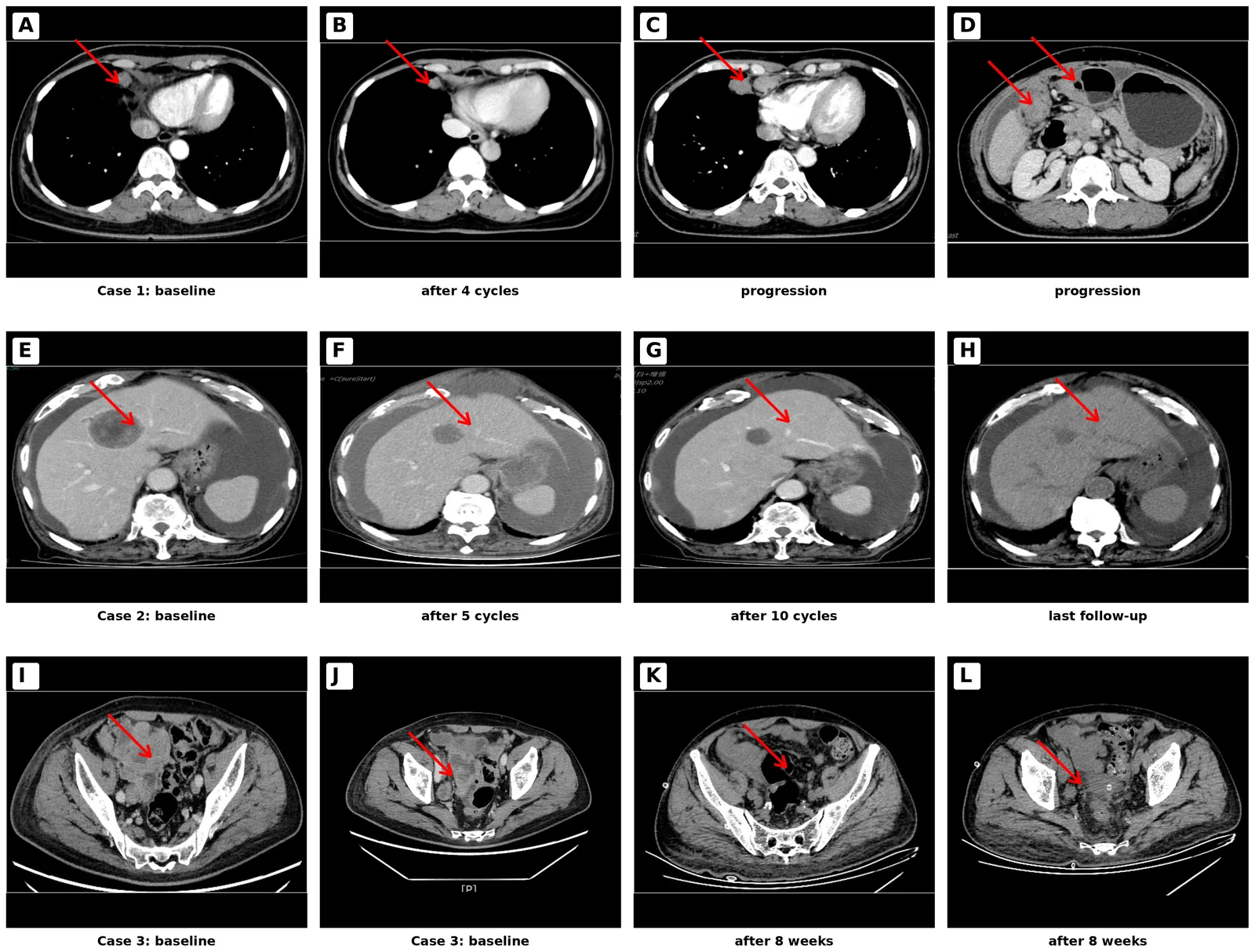
Serial CT findings before and after treatment in the three patients. **(A–D)** Case 1: cardiophrenic and peritoneal lesions at baseline, after four treatment cycles, and at progression. **(E–H)** Case 2: hepatic lesions at baseline, after five cycles, after 10 cycles, and at the most recent follow-up in February 2026. **(I–L)** Case 3: two representative abdominopelvic levels at baseline and after approximately 8 weeks of QL1706 treatment. Red arrows indicate representative lesions.

Extensive peritoneal disease remained after locoregional treatment, and the patient declined conventional cytotoxic chemotherapy. Her Eastern Cooperative Oncology Group performance status (ECOG PS) was 1, and she had no major comorbidities. Following multidisciplinary discussion, QL1706 5 mg/kg plus bevacizumab 7.5 mg/kg was administered intravenously every 3 weeks because there were no clear contraindications to immune checkpoint or anti-vascular endothelial growth factor (VEGF) therapy. Treatment began on February 28, 2025. Baseline CT showed peritoneal disease and multiple right cardiophrenic angle nodules considered radiologically consistent with metastatic lymph nodes; the largest long-axis diameter was approximately 11 mm (**Figure 2A**). After four cycles, the lesions had decreased slightly or remained essentially unchanged (**Figure 2B**). After cycle 11, the peritoneal, mesenteric, and omental disease progressed, ascites increased, and the cardiophrenic nodules enlarged (**Figures 2C, D**). The best response was stable disease (SD), with a progression-free survival (PFS) of approximately 7 months. Abdominal distension and pain improved during treatment, and no grade 3 or higher treatment-related adverse events were recorded.

### 2.2 Case 2

An 89-year-old man presented with abdominal pain, a palpable abdominal mass, massive ascites, and hypoalbuminemia. Cytopathologic examination of ascitic-fluid cell-block specimens showed malignant epithelioid cells (**Figures 1C, D**). The cells were positive for calretinin, D2-40, WT1, CK7, and pancytokeratin, with focal CK5/6 expression, and were negative for CEA, CDX2, and CK20. The integrated morphologic and immunophenotypic findings supported a diagnosis of EMPM. The PD-L1 CPS was 0, and no additional tumor molecular results were available in the medical record. The patient did not undergo surgery; therefore, PCI was not assessed. He had received no previous systemic anticancer therapy.

The patient had an ECOG PS of 2, hypertension, massive ascites, and hypoalbuminemia and was considered unsuitable for CRS-HIPEC or intensive cytotoxic chemotherapy. After multidisciplinary discussion, a chemotherapy-free regimen of intravenous QL1706 5 mg/kg every 3 weeks plus oral lenvatinib was selected. Given his advanced age, frailty, and comorbidities, lenvatinib was started at 4 mg/day and increased to 8 mg/day after initial tolerability was confirmed.

Treatment began on April 9, 2025. Baseline CT demonstrated a 53 × 50-mm lesion in the left hepatic lobe, extensive peritoneal, omental, and mesenteric involvement with omental caking, and massive ascites (**Figure 2E**). After five cycles, the hepatic lesion had decreased to approximately 28 × 26 mm and the omental nodules had markedly regressed, meeting the criteria for a partial response (PR) (**Figure 2F**). The PR was maintained after 10 cycles (**Figure 2G**), and CT in February 2026 showed no definite progression (**Figure 2H**). Ascites nevertheless fluctuated in the setting of persistent hypoalbuminemia and widespread serosal involvement. A mild infusion-related reaction resolved with symptomatic management and did not affect subsequent treatment. At the most recent follow-up in February 2026, PFS exceeded 10 months; the patient remained in PR and continued the same regimen.

### 2.3 Case 3

A 70-year-old man presented with massive ascites, bilateral lower-extremity edema, severe hypoalbuminemia, and nephrotic-range proteinuria. Image-guided core-needle biopsy of an abdominal mass showed malignant epithelioid tumor cells (**Figures 1E, F**). The tumor cells were positive for calretinin, D2-40, WT1, CK7, pancytokeratin, and vimentin and negative for CEA, SATB2, CK20, CD31, and CD34. The Ki-67 proliferation index was approximately 25% in hotspot areas. The morphologic and immunophenotypic findings supported a diagnosis of EMPM. The PD-L1 CPS was 55. No additional tumor molecular testing was performed. The patient did not undergo surgical exploration, and PCI was therefore not assessed.

Before immunotherapy, 24-hour urinary protein excretion was 9.83 g, serum albumin was 17.7 g/L, and serum creatinine was 55 μmol/L. Nephrology consultation established the presence of nephrotic syndrome on the basis of heavy proteinuria, hypoalbuminemia, and edema, but the underlying cause remained uncertain because kidney biopsy was not performed. Systemic therapy was required because of diffuse disease and anticipated poor tolerance of chemotherapy. Severe baseline proteinuria and hypoalbuminemia were considered to increase the renal risk of antiangiogenic therapy; therefore, no antiangiogenic agent was added. After multidisciplinary discussion, QL1706 monotherapy at 5 mg/kg intravenously every 3 weeks was initiated together with supportive renal management.

QL1706 was started on August 1, 2025. Baseline CT on July 1, 2025 showed multiple peritoneal and mesenteric masses, the largest measuring approximately 97 × 65 mm, with areas of necrosis (**Figures 2I, J**). After approximately 8 weeks of treatment, the dominant lesion had decreased to approximately 67 × 32 mm, and the treating team classified the response as PR according to RECIST 1.1 (**Figures 2K, L**). Proteinuria and edema worsened during treatment. Because nephrotic syndrome was already present before QL1706, it could not be regarded as a newly developed treatment-related syndrome; however, without renal histology, an immune-mediated contribution to the subsequent deterioration could not be excluded. QL1706 was discontinued after multidisciplinary reassessment. The patient was subsequently hospitalized at a local hospital, and cross-institutional follow-up limitations prevented us from obtaining serial quantitative measurements of urinary protein, serum albumin, and renal function. Follow-up brain magnetic resonance imaging identified a new intracranial lesion radiologically suspicious for metastasis, without pathologic confirmation. Overall PFS was approximately 4 months.

The diagnostic basis, baseline disease characteristics, treatment selection, and outcomes of the three patients are summarized in **Table 1**. Radiographic response was assessed by the treating multidisciplinary team on serial CT using RECIST 1.1 as the evaluation framework; no blinded independent imaging review was performed.

**Table 1:** Diagnostic characteristics, treatment selection, and clinical outcomes of the three patients with EMPM.

Feature	Case 1	Case 2	Case 3
Age/sex	53 years/female	89 years/male	70 years/male
ECOG PS	1	2	1
Diagnostic specimen	Resected pelvic/peritoneal lesions	Ascitic-fluid cell-block specimens	Image-guided core-needle biopsy of an abdominal mass
Key pathology/immunophenotype	Calretinin+, CK5/6+, D2-40+, CK7+; PAX8-, ER-, PR-, SATB2-, CDX2-; Ki-67 approximately 10%-15%	Calretinin+, D2-40+, WT1+, CK7+, pan-CK+; focal CK5/6+; CEA-, CDX2-, CK20-; Ki-67 approximately 10%	Calretinin+, D2-40+, WT1+, CK7+, pan-CK+, vimentin+; CEA-, SATB2-, CK20-, CD31-, CD34-; Ki-67 approximately 25% in hotspot areas
PD-L1 CPS	10	0	55
Additional molecular testing	No testing beyond PD-L1	No additional results available in the medical record	No testing beyond PD-L1
Disease extent/PCI/CC	Large-volume ascites, numerous miliary peritoneal implants, and diffuse omental involvement; PCI not recorded; formal CRS not performed, so CC not applicable	Unresectable extensive peritoneal, omental, and mesenteric disease, massive ascites, and a hepatic lesion; no surgery, PCI not assessed	Unresectable diffuse peritoneal and mesenteric masses, largest 97 × 65 mm; no surgery, PCI not assessed
Previous anticancer treatment	Gynecologic surgery; repeat laparoscopy with adhesiolysis and catheter placement; three sessions of postoperative catheter-based hyperthermic intraperitoneal chemotherapy; no previous systemic therapy	None	None
QL1706-based regimen	QL1706 5 mg/kg + bevacizumab 7.5 mg/kg, every 3 weeks	QL1706 5 mg/kg every 3 weeks + lenvatinib 4 mg/day, increased to 8 mg/day	QL1706 5 mg/kg every 3 weeks, monotherapy
Rationale for treatment selection	Extensive peritoneal disease remained after locoregional treatment; patient declined cytotoxic chemotherapy; no clear contraindication to anti-VEGF therapy	Advanced age, ECOG PS 2, massive ascites, and hypoalbuminemia; dose-reduced chemotherapy-free combination selected	Anticipated poor chemotherapy tolerance; severe baseline proteinuria/hypoalbuminemia; antiangiogenic therapy avoided
Best response	SD	PR	PR
PFS/treatment status	Approximately 7 months; discontinued after progression	>10 months; ongoing PR and treatment at last follow-up	Approximately 4 months; discontinued for worsening nephrotic syndrome, followed by progression
Main safety findings	No grade 3 or higher treatment-related adverse events	Mild infusion-related reaction that resolved with treatment	Worsening pre-existing proteinuria, edema, and hypoalbuminemia; an immune-mediated contribution could not be excluded
Status at last follow-up	Progression of peritoneal and cardiophrenic disease	Ongoing PR	Disease progression with a radiologically suspected brain metastasis

## 3 Discussion

All three patients had limited access to standard locoregional treatment or intensive chemotherapy because of disease extent, age, performance status, or comorbidities. QL1706-based therapy produced PR in two patients and approximately 7 months of SD in one. These three clinical courses - a durable response, rapid tumor regression followed by treatment discontinuation, and disease stabilization with symptomatic improvement - show why the value of QL1706 in EMPM cannot be captured by a single imaging time point. Tumor sensitivity, the contribution of the combination strategy, and the ability to remain on treatment all shaped the observed benefit.

The evidence base for systemic therapy in EMPM still derives largely from pleural mesothelioma studies and small peritoneal mesothelioma series. CheckMate 743 and IND.227 established that immune checkpoint inhibition can improve outcomes in unresectable pleural mesothelioma (3, 4), while retrospective cohorts, the atezolizumab-bevacizumab study, and recent real-world data have documented durable responses in peritoneal mesothelioma (5–8). QL1706 provides fixed-ratio PD-1 and CTLA-4 blockade while limiting systemic exposure to the CTLA-4 component (9–11). Although no direct comparison with established dual-checkpoint regimens is available, the marked early regression observed in Case 3 without an antiangiogenic partner provides a clinical observation of QL1706 monotherapy activity in EMPM.

Case 2 extends this signal in a different clinical setting. Despite being 89 years old, having a PD-L1 CPS of 0, and presenting with massive ascites and hypoalbuminemia, the patient maintained PR for more than 10 months with QL1706 plus lenvatinib. For older patients unable to tolerate cytotoxic chemotherapy, this course suggests that dual-checkpoint blockade combined with VEGF-pathway inhibition may offer a practicable chemotherapy-free approach. Beyond direct inhibition of angiogenesis, VEGF blockade may improve abnormal tumor vasculature and facilitate immune-cell access to the tumor microenvironment (12); the reported activity of atezolizumab plus bevacizumab in peritoneal mesothelioma also supports this direction (6). Case 1, in which QL1706 plus bevacizumab achieved symptomatic improvement and approximately 7 months of disease control, represents another clinical context for this strategy. These cases cannot separate the individual contributions of QL1706 and the antiangiogenic agents, but they raise a clinically relevant question: can VEGF-pathway inhibition deepen or prolong response, or make immunotherapy feasible for patients otherwise unable to receive chemotherapy?

Case 3 illustrates that tumor sensitivity does not necessarily translate into durable clinical benefit. The patient achieved PR with QL1706 monotherapy but discontinued treatment after worsening proteinuria and edema in the setting of pre-existing nephrotic syndrome. Nephrology consultation had established the syndrome before immunotherapy, yet the underlying renal disease and the contribution of immune therapy to subsequent deterioration could not be determined without kidney biopsy; serial quantitative data were also unavailable after the patient was hospitalized locally. The central point is not to retrospectively assign a renal adverse event, but to recognize that baseline organ function may determine whether an active treatment can be sustained. In patients with severe proteinuria or hypoalbuminemia, renal assessment should directly inform treatment selection rather than serving only for *post hoc* toxicity attribution.

The PD-L1 CPS values in the three patients were 10, 0, and 55, and the outcomes did not improve with increasing expression. The most durable response occurred in the patient with a CPS of 0, whereas the patient with a CPS of 55 discontinued treatment because of worsening comorbidity. PD-L1 reflects expression at a particular site and time and cannot capture immune-cell infiltration, angiogenic signaling, or spatial heterogeneity across peritoneal lesions. In this series, PD-L1 status would not have been a suitable basis for excluding QL1706 therapy. The diffuse growth pattern of EMPM also complicates response assessment: peritoneal thickening, omental caking, and ascites may not be adequately represented by a small number of target lesions, and ascites is additionally influenced by hypoalbuminemia and renal disease. RECIST 1.1 therefore provides a useful framework, but findings should be interpreted together with the overall extent of peritoneal disease, symptoms, and treatment duration.

This study is limited by its small sample size, heterogeneous treatment regimens, absence of independent imaging review, limited molecular data, and incomplete follow-up information from the outside hospital for Case 3. The prior treatment in Case 1 consisted of surgery followed by postoperative catheter-based hyperthermic intraperitoneal chemotherapy rather than standard CRS-HIPEC; consequently, a CC score could not be used to characterize the outcome of that locoregional treatment. These limitations preclude comparison of monotherapy with combination therapy, but they do not erase a clinical signal that warrants further study: in patients with EMPM and limited conventional options, QL1706 alone or with antiangiogenic treatment can produce clinically meaningful disease control. Future studies should address three linked questions: which tumors are genuinely sensitive to dual-checkpoint blockade, whether VEGF-pathway inhibition can extend that sensitivity, and how baseline organ function influences treatment durability.

## 4 Conclusion

QL1706-based therapy achieved disease control in all three patients with EMPM who had limited conventional treatment options, including a partial response lasting more than 10 months in an elderly patient with PD-L1-negative disease and rapid tumor regression with QL1706 monotherapy. The different clinical courses suggest that overall benefit depends on tumor sensitivity, the accompanying treatment strategy, and the patient’s ability to remain on therapy. The role of QL1706, alone or combined with antiangiogenic treatment, should be evaluated in larger studies using more consistent treatment approaches.

## Data Availability

The original contributions presented in the study are included in the article/supplementary material. Further inquiries can be directed to the corresponding author.
